# Evaluating Drought Tolerance in *Codonopsis pilosula* Seedlings: Combining Growth, Physiology, Yield, and Tolerance Indices

**DOI:** 10.3390/ijms26041600

**Published:** 2025-02-13

**Authors:** Hongyan Wang, Yuan Chen, Fengxia Guo, Di Wu, Wei Liang, Pengbin Dong, Jiali Cheng

**Affiliations:** College of Agronomy, College of Life Science and Technology, State Key Laboratory of Aridland Crop Science, Gansu Agricultural University, Lanzhou 730070, China; why8852@163.com (H.W.); guofx@gsau.edu.cn (F.G.); andywu173@163.com (D.W.); liangw@gsau.edu.cn (W.L.); dongpb@stumail.nwu.edu.cn (P.D.); chengjiali24@icloud.com (J.C.)

**Keywords:** *Codonopsis pilosula*, drought stress, growth-physiology-yield composite index, seedlings, hole sowing

## Abstract

Drought stress during the *Codonopsis pilosula* (Campanulaceae) seedling stage significantly affects its growth, quality, and yield. The aim of this study was to identify drought-tolerant cultivars of *C. pilosula* by using the growth—physiology—yield composite index (GPYCI) and drought-tolerant indices. Nine *C. pilosula* cultivars were evaluated under normal-watered (black plastic film hole sowing, BF) and water-stressed (spread in the open field, SF) conditions in a design that adopted a two-factor paired experiment with three replications. The emergence rate was significantly influenced by the water treatment, while both the water treatment and the cultivar affected root length, proline content, APX activity, and chlorophyll levels. The G1 cultivar performed better than others in multiple aspects. Yields and their attributes varied among cultivars under different water levels. The average yield was 7350.76 kg/hm^2^ under BF conditions and 4856.32 kg/hm^2^ under SF conditions. Drought stress reduced the total root length, single root fresh weight, and yield by 18.33%, 28.4%, and 33.9%, respectively. Correlation analysis revealed unique physiological response mechanisms to water changes among cultivars. Drought tolerance indices and comprehensive factor analysis indicated varying levels of drought tolerance among cultivars. This study has provided valuable insights into the growth, physiology, and yield response of *C. pilosula* under drought conditions and laid the foundation for breeding drought-tolerant cultivars.

## 1. Introduction

Climate change, a concern affecting everyone, has brought many challenging tasks to modern agriculture [1]. The rising temperature and fluctuating precipitation caused by climate change have been widely reported to intensify the frequency and severity of drought on a global scale [1,2]. Drought-induced agricultural losses account for approximately 70% of the global potential yield losses each year [3]. *Codonopsis pilosula*, a traditional herbal plant, and its dry roots, known as *Codonopsis radix* or Dangshen [4], can be used for both food and medicine [5] and are widely used in Asian countries.

Gansu Province is the regional production area of *C. pilosula* [6]. It features a dry climate, with 75% of its total area being arid or semi-arid regions. Seasonal drought and drought during the critical growth period severely hinder the quality and yield of *Codonopsis radix* [7]. Breeding drought-tolerant new *C. pilosula* cultivars is a crucial strategy to solve this issue. *C. pilosula* reproduces via seeds [8], and the yield and quality of *Codonopsis radix* are largely dependent on the quality of its seedlings [9]. *Codonopsis* seedlings demand more water than during the stage of medicinal value formation of *C. pilosula*, mainly because water deprivation causes a decrease in water potential, nutrient uptake, and photosynthesis, as well as induces oxidative damage from reactive oxygen species (ROS) and the disturbance of metabolism, finally resulting in a reduced production and yield quality of *Codonopsis* seedlings [10,11,12]. However, plant responses in terms of leaf water relations, stomatal regulation, photosynthesis, and other regulatory processes vary among species and cultivars [13]. For example, Zhang et al. [14] suggests that water stress reduces the stomatal conductance of field-grown maize through osmotic adjustment, thereby improving water use efficiency. Shi et al. [15] suggests that a higher rate of soluble sugar accumulation in the source is one of the reasons for triggering non-sequential leaf senescence. A higher rate of soluble sugar mobilization during non-sequential senescence contributes to high and stable wheat yield and drought tolerance. At the same time, there were significant differences and a negative correlation in stomatal density and size between indica and japonica rice [16]. The growth–physiology–yield comprehensive index (GPYCI) is an important reference for enhancing drought resistance as it is related to the plant’s adaptive mechanisms under stress conditions [17].

Identifying traits that tolerate drought stress can help breeders develop drought-resistant plant types. Hafeez et al. [18] believe that in the drought-tolerant breeding of wheat, selecting plants with excellent traits such as efficient water use and deep root systems is of great significance for improving breeding strategies. Toulotte et al. [19] believe that utilizing the rich genetic diversity of wild crop ancestors is a promising way. These wild relatives can thrive in diverse environments and have many adaptive traits. Identifying and introducing these traits into staple cereals can cut yield losses under stress. Through the evaluation of 114 rice genotypes under water stress, Verma and Sarma [20] found significant variations in the root and shoot traits of each genotype. Eleven significant marker—trait associations and a new QTL related to root length associated with RM127 were detected. The marker alleles related to these can be used in marker-assisted selection to improve root and drought tolerance traits. Although *C. pilosula* exhibits high genetic diversity, studies assessing the genotypes of the plant under drought stress using the GPYCI and drought index are relatively scarce. Selecting the best-performing cultivars from under well-watered and water-stressed conditions is an effective method for identifying drought-resistant genotypes [21]. Several drought tolerance indices have been proposed in other research [1,22,23], most of which could be used in this study to select the best *C. pilosula* cultivars for drought tolerance. We hypothesized that the responses of *C. pilosula* cultivars vary significantly under different water levels, and that the best performing cultivar can be chosen using the growth-physiology-yield composite index (GPYCI) and drought tolerance indices. Therefore, the present work was aimed at evaluating the GPYCI of the seedlings in different *C. pilosula* cultivars under well-watered and water-stressed conditions and identifying the best drought-tolerant cultivar using drought tolerance indices.

## 2. Results

### 2.1. Variations in Stress-Resistant Physiological Indicators of Different C. pilosula Cultivar Seedlings

**Figure 1:**
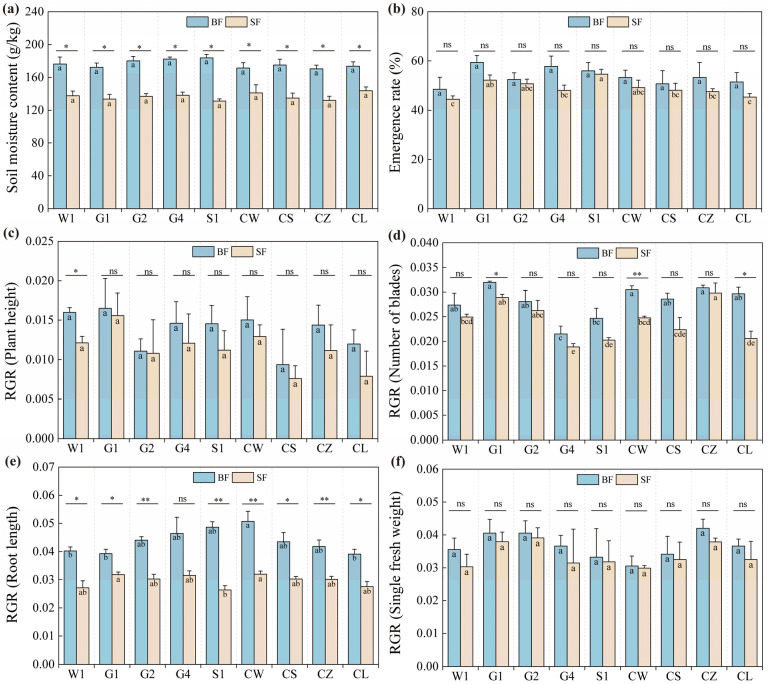
Growth indicators of C. pilosula cultivar seedlings. The data in the figure represent the $\bar{X}$ ± SD (standard deviation) (r = 3). (**a**) Soil moisture content; (**b**) emergence rate; (**c**) RGR (plant height); (**d**) RGR (number of blades); (**e**) RGR (root length); (**f**) RGR (single fresh weight); Different lowercase letters mean a significant difference (*p* < 0.05); ‘*’ indicates a significant difference (*p* < 0.05); ‘**’ indicates a highly significant difference (*p* < 0.01); ‘ns’ indicates that the difference is not significant.

As shown in the soil measurements on July 30 (Figure 1a), the average soil moisture content in the open field plots (SF) for each treatment was 136.5 g/kg. Given the preference of *Codonopsis pilosula* seedlings for humidity, this moisture level might slow down their growth, potentially limiting plant height and root length. In contrast, the soil water content in the black plastic film hole-sown plots (BF) was approximately 176.1 g/kg, which could foster better growth and development of *C. pilosula*, potentially increasing yield and quality. Thus, the soil conditions in this study are suitable for assessing the drought resistance of various *C. pilosula* cultivars.

Figure 1 illustrates significant trait variations among *C. pilosula* cultivars under BF and SF conditions. Under the BF condition, the emergence rate did not differ significantly among the cultivars, with the G1 cultivar showing the highest rate. However, under the SF condition, significant differences were observed, particularly with the S1 and G1 cultivars having higher emergence rates than the W1 cultivar (Figure 1b). The analysis of variance indicated that the water treatment significantly affected the emergence rate, RGR (plant height), RGR (number of blades), and RGR (root length), but the cultivar only significantly affected RGR (number of blades) and has an interaction effect on RGR (single fresh weight) (Table 1). For the relative growth rate of the number of leaves, both the water treatment and the cultivar had significant effects. Under the BF condition, the G1 cultivar had the highest rate, while the G4 cultivar was significantly lower than the W1 cultivar. Under the SF condition, the CZ cultivar exhibited the highest relative growth rate, with the G4 cultivar again being significantly lower than the W1 cultivar (Figure 1d). The water treatment significantly influenced the emergence rate, the relative growth rate of plant height, and the root length of *C. pilosula* seedlings. Both the water treatment and the cultivar significantly influenced the relative growth rate of the number of blades (Table 1). Under the BF condition, the CW cultivar had the highest rate, but water stress impeded root growth. Under the SF condition, the G1 cultivar showed the highest relative growth rate, indicating better water and nutrient uptake through root development, which is crucial for drought tolerance (Figure 1e).

**Figure 2:**
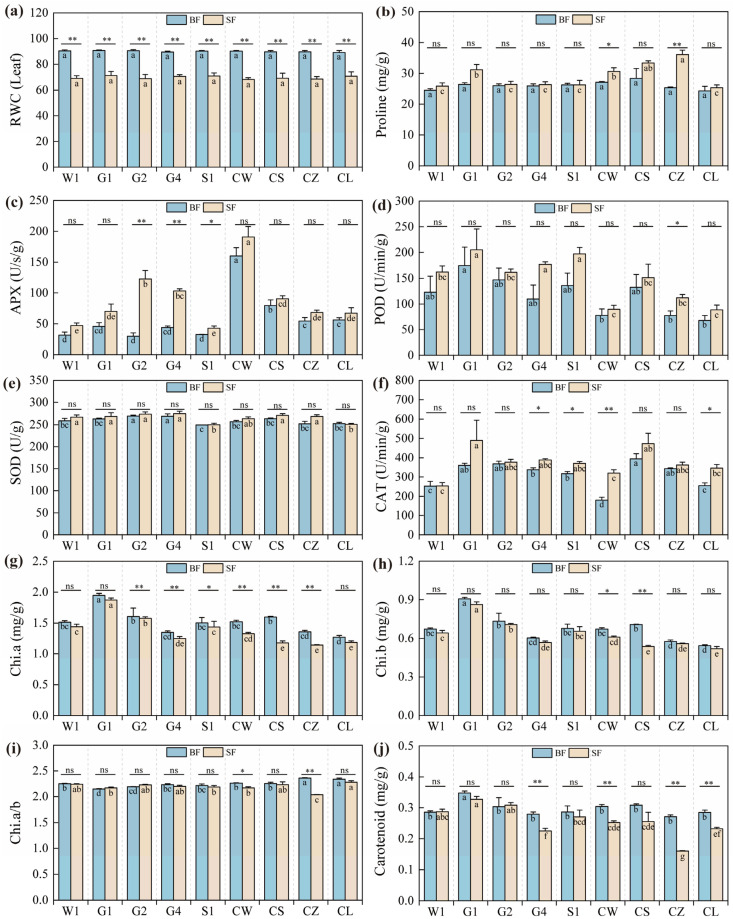
Physiological indicator responses of *C. pilosula* cultivar seedlings. The data in the figure represent the $\bar{X}$ ± SD (r = 3). (**a**) Relative water content of leaf (RWC (leaf)); (**b**) leaf proline content; (**c**) leaf ascorbate peroxidase (APX) content; (**d**) leaf peroxidase (POD) content; (**e**) leaf superoxide dismutase (SOD) content; (**f**) leaf catalase (CAT) content; (**g**) content of chlorophyll a (Chi.a); (**h**) content of chlorophyll b (Chi.b); (**i**) content of chlorophyll a/b (Chi.a/b); (**j**) content of carotenoids; Different lowercase letters mean a significant difference (*p* < 0.05); ‘*’ indicates a significant difference (*p* < 0.05); ‘**’ indicates a highly significant difference (*p* < 0.01); ‘ns’ indicates that the difference is not significant.

Water stress significantly impacts the rate and efficiency of various physiological and biochemical traits by disrupting physiological pathways. Significant alterations were observed in the relative water content (RWC) of leaves, proline content, antioxidant enzyme activities (such as superoxide dismutase (SOD), catalase (CAT), peroxidase (POD), and ascorbate peroxidase (APX)), as well as the chlorophyll content, which were influenced by both the cultivar and the water conditions (Figure 2, Table 2). The cultivars had no significant effect on the RWC of leaves (Figure 2a, Table 2), but they did influence other physiological indicators and had no interaction effect on RGR (Leaf), POD, SOD, and CAT. Under the SF condition, the proline content notably increased in the CZ, CS, CW, and G1 cultivars compared to the W1 cultivar (Figure 2b). The CW cultivar exhibited the highest activity of the APX enzyme before and after stress, while the G2 cultivar showed the most significant change in APX activity (Figure 2c). Under the SF condition, the G1 cultivar had the highest activities of CAT and POD, and there was no significant difference in SOD activity among the cultivars under both treatments (Figure 2d–f). The G1 cultivar had the highest levels of chlorophylls a and b, as well as carotenoid (Figure 2g–j), suggesting enhanced photoprotection under the SF condition. In contrast, the CZ cultivar showed the most significant changes in chlorophyll a, the chlorophyll a/b ratio, and carotenoid, indicating a strong physiological response to water stress, which affected pigment synthesis, degradation, or regulation. The a/b ratio increased in the G1 and G2 cultivars under the SF condition compared to the BF condition (Figure 2i), likely due to a slight rise in chlorophyll a, which could boost carbon fixation activity. This suggests that the drought stress was not severe enough to markedly inhibit photosynthesis in the G1 and G2 cultivars.

### 2.2. Yield and Its Attributes of C. pilosula Cultivar Seedlings

**Figure 3:**
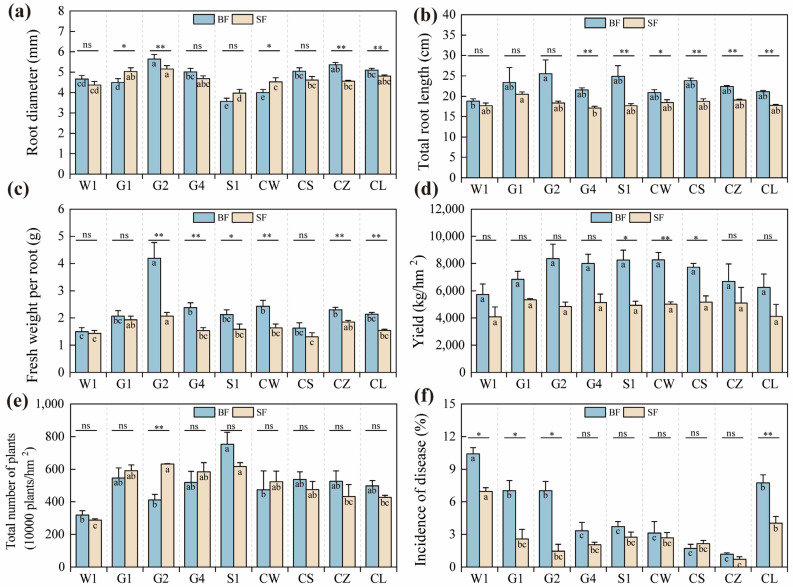
Yield, and yield-related indicators of *C. pilosula* cultivar seedlings. The data in the figure represent the $\bar{X}$ ± SD (r = 3). (**a**) Root diameter; (**b**) total root length; (**c**) fresh weight per root; (**d**) fresh yield; (**e**) total number of plants; (**f**) incidence of disease; Different lowercase letters mean a significant difference (*p* < 0.05); ‘*’ indicates a significant difference (*p* < 0.05); ‘**’ indicates a highly significant difference (*p* < 0.01); ‘ns’ indicates that the difference is not significant.

Significant differences (*p* < 0.05, Figure 3, Table 3) were exhibited by *C. pilosula* cultivars in biomass production, yield, and yield-related traits under two water levels. The analysis of variance showed that the water treatment had a significant impact on the total root length, fresh weight per root, yield, and disease incidence. Meanwhile, the cultivar significantly influenced the root diameter, fresh weight per root, total number of plants, and disease incidence. Notably, there was an interaction effect between the water treatment and the cultivar on the root diameter, fresh weight per root, and disease incidence (Table 3). Under the BF and SF conditions, the mean total root lengths were 22.47 cm and 18.35 cm, respectively. The corresponding average single root fresh weights were 2.31 g and 1.65 g, and the average yields were 7350.76 kg/hm^2^ and 4856.32 kg/hm^2^. Compared to the BF condition, water stress reduced the total root length, single root fresh weight, and yield by 18.33%, 28.4%, and 33.9%, respectively. Under the BF and SF conditions, the G2 cultivar had the thickest root diameter, which was 5.64 mm and 5.15 mm, respectively (Figure 3a). Under the same conditions, the G1 cultivar had the longest total root length, measuring 23.36 cm and 20.46 cm, respectively (Figure 3b). The G2 cultivar also had the highest single root fresh weight, being 4.20 g and 2.06 g, respectively, under the BF and SF conditions, with extremely significant differences shown (Figure 3c). The yield of the G2 cultivar was the highest, reaching 8366.00 kg/hm^2^ and 4834.68 kg/hm^2^ under the BF and SF conditions, respectively (Figure 3d). Under the BF and SF conditions, the S1 cultivar had the highest plant count, which was 752.50 × 10^4^ and 615.93 × 10^4^ plants/hm^2^, respectively (Figure 3e). Under the BF and SF conditions, the W1 cultivar had the highest incidence rate, being 10.4% and 7.0%, respectively, while the CZ cultivar had the lowest, with rates of 1.2% and 0.7%, respectively (Figure 3f).

### 2.3. Correlation Analysis of C. pilosula Cultivar Seedlings

**Figure 4:**
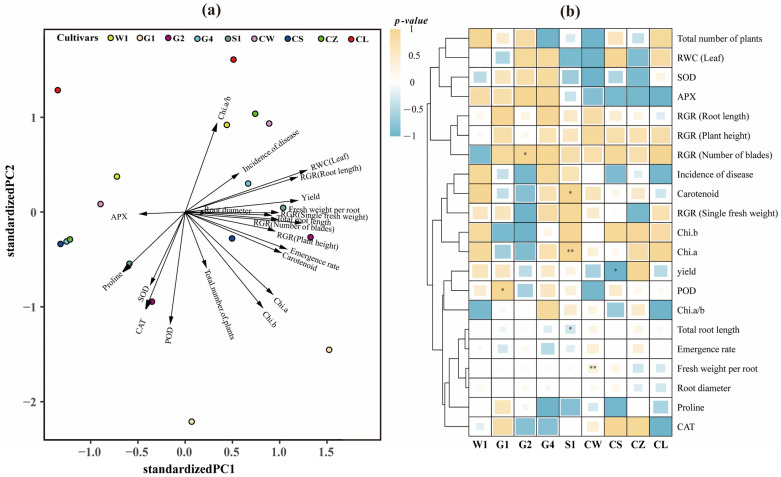
Biplot analysis and Pearson’s correlation analysis of the GPYCI for *C. pilosula* seedlings under the BF and SF conditions. (**a**) Biplot analysis of indices for *C. pilosula* cultivars grown under the BF and SF conditions; The length of the variable vector reflects the contribution degree of the variable to the principal component, while the angle between the vectors indicates the correlation between the variables. (**b**) Pearson’s correlation analysis of the GPYCI of *C. pilosula* seedlings under the BF and SF conditions. The size of each square represents the magnitude of the Pearson correlation coefficient between variables and cultivars under different treatments, and different colors represent the significance level. GPYCI is the growth–physiology–yield composite index. ‘*’ indicates a significant difference (*p* < 0.05); ‘**’ indicates a highly significant difference (*p* < 0.01).

From the distribution of the selected growth–physiology–yield composite index (GPYCI) and cultivars on the PC1 axis in the principal component biplot (Figure 4a), the growth-related indicators (e.g., relative growth rate-related indices) and some physiological indicators (e.g., antioxidant enzymes and chlorophyll) exhibit different distribution patterns on the PC1 axis, suggesting that the PC1 axis reflects a comprehensive performance trend of plants during growth and environmental adaptation. However, it can be observed that from the left to the right on the PC1 axis, the distribution of different cultivars is relatively dispersed. Cultivar CL is located at the far left of the PC1 axis, while cultivar G1 is at the far right. This implies that there are differences in the physiological responses of different cultivars to drought. It can also be seen from Figure 4b that different cultivars have differences in tolerance to different GPYCIs. Among them, the peroxidase (POD) activity of the G1 cultivar shows a significant positive correlation under the two water conditions; the relative water content (RWC) of leaves of the G2 cultivar shows a significant positive correlation under the two water conditions; the chlorophyll a (Chi.a) and carotenoids of the S1 cultivar show a significant positive correlation under the two water conditions, while the total number of plants per unit area shows a significant negative correlation; the fresh weight of a single root of the CW cultivar shows a significant positive correlation under the two water conditions; and the yield of the CS cultivar shows a significant negative correlation under the two water conditions. This indicates that the genetic characteristics of *C. pilosula* cultivars play a key role in responding to water changes. Each cultivar has its own unique physiological response mechanism, which determines their performance of various indices under different water environments.

### 2.4. Drought Tolerance Indices and Comprehensive Factor Analysis

Drought tolerance indices can be utilized to assess the differences in drought tolerance among cultivars, the sensitivity of cultivars to drought, as well as the stability and adaptability of cultivars under drought conditions. In this study, we calculated the drought tolerance indices through physiological and yield indicators under two water levels to evaluate the drought resistance of different *C. pilosula* cultivars at the seedling stage (Table 4). The higher the values of SSI% and SSI reduction, the more sensitive the cultivar is to stress. Meanwhile, larger values or values closer to one for other indices indicate that the cultivar is more physiologically or yield-stable under the SF condition and is more drought-tolerant. From the results of the drought tolerance indices, it can be observed that when different cultivars are evaluated for drought tolerance using different drought tolerance indices, the results of drought tolerance vary. Therefore, it is necessary to comprehensively consider various drought tolerance indicators for the evaluation of the drought resistance of *C. pilosula* seedling cultivars. Based on the principal component analysis of 22 drought resistance indices, the eigenvalues of the first five principal components were greater than one. The contribution rates were 38.76%, 23.52%, 11.72%, 10.39%, and 7.46%, respectively, and the cumulative contribution rate was 91.86%. Consequently, the eigenvalues and contribution rates of the first five principal components (Table A1) were extracted to calculate the weight values of each index (Table A2). According to the test indices’ membership degree, weight value, and the addition and multiplication operations of all the indices, the comprehensive evaluation indices ranked the cultivars in the order of G1 > CZ > CS > G2 > S1 > CW > G4 > CL > W1 (Table 5).

## 3. Discussion

The growth of *Codonopsis pilosula*, especially in the seedling stage, is frequently hampered by various abiotic stress factors, such as temperature, precipitation, light intensity, and heavy metal [24,25], with drought being a major concern. Drought refers to a climatic phenomenon in which there is no rain for a long period or the precipitation is abnormally low, resulting in dry air and water shortage in the soil, thus having an adverse impact on plants [26]. Recently, researchers have cloned hundreds of genes related to crop drought resistance through genetic and molecular methods, but only a few have been used in breeding, showing that drought resistance regulation is complex [27]. Numerous studies [28,29] have also elucidated the drought resistance mechanism of plants. However, there remain challenges in applying these findings to large-scale breeding programs. For instance, how to effectively introduce such genetic modifications into plant varieties without compromising other important agronomic traits and ensure the stable inheritance and expression of these genes across different varieties. Consequently, identifying the key genotype and assessing its performance under different water stress conditions are crucial objectives for plant researchers [30]. Under natural conditions of water stress, evaluating various growth, physiological, and yield aspects of *C. pilosula* cultivars offers breeders an efficient approach. These trait evaluations can serve as the basis for selecting drought-tolerant materials, facilitating their promotion in drought-tolerant areas or the development of new drought-tolerant *C. pilosula* cultivars [31].

### 3.1. Drought Stress Affects the Growth Rate of C. pilosula Seedlings

Drought is a major abiotic stress that has numerous adverse effects on crop growth and development [32]. It mainly manifests itself by slowing down the growth rate of crop stems, leaves, and roots. While stems and leaves are crucial for sunlight absorption, competition for space, and biomass accumulation [33], leaves are the main site of photosynthesis in plants [34,35,36], and the root system facilitates the plant’s water and nutrient uptake [37,38]. In this study, under the water-stress condition (SF), the emergence rate and the relative growth rates of plant height, as well as the number of leaves, root length, and the fresh weight of individual roots of *C. pilosula* seedlings generally showed a reduction compared to the normal-watered condition (BF). Makonya et al. [39] observed a decrease in growth rates and biomass accumulation in two chickpea cultivars subjected to drought during their vegetative and flowering stages, and this was consistent with the results of this study. The reduction in the relative growth rate of growth indicators can be attributed to the reallocation of resources towards stress tolerance mechanisms rather than growth and development [40]. Among them, cultivars G1, G2, and CZ exhibited relatively better growth under drought conditions, with higher emergence rates and growth rates in plant height, the number of leaves, root length, and the fresh weight of individual roots. This is because they were selected under extremely arid conditions, and drought hardening can improve the drought tolerance and adaptability of plants [32].

### 3.2. Drought Stress Affects Physiological Changes in C. pilosula Seedlings

Under drought stress, the physiological responses of different cultivars are the key indicators of how plants tolerate drought stress [41] as they reflect a plant’s ability to maintain cellular homeostasis, prevent oxidative damage, and regulate water absorption and loss [42,43]. Maintaining a good water status is crucial for optimal physiological function and growth. Some studies have shown that a high relative water content (RWC) is closely related to drought resistance [44,45]. In this study, under the BF condition, most cultivars maintained relatively high RWC values. However, under the SF condition, the RWC significantly decreased across all cultivars, and the degree of reduction varied. Higher RWC indicates better maintenance of water balance and potentially greater drought tolerance, as plants can maintain turgor pressure and normal physiological functions [46]. Cultivar W1 showed the most significant reduction in RWC, suggesting that it is more susceptible to water stress when maintaining tissue water balance. Osmotic adjustment is regarded as an important component of drought tolerance. As an osmoregulatory substance, proline accumulates in large quantities when plants are subjected to environmental stresses such as drought. It can increase the solute concentration of the cell and reduce the osmotic potential of the cell, thus enabling the cell to absorb more water from the external environment, maintaining the turgor pressure of the cell, and ensuring the normal physiological function of the cell [47,48]. In this study, the proline content of all cultivars increased under the SF conditions, but the extent of the increase varied. Cultivars CW and CZ showed a significant increase in proline content, suggesting a stronger osmoprotective response. In addition, the initial proline content of different cultivars may affect the extent of their increase under the SF conditions. Cultivars with lower basal proline content may have more scope for up-regulation. Salsinha et al. [49] showed that proline content, which is usually elevated under drought stress, acts as an osmoprotectant, helping to maintain cellular turgor and protect cellular structures. Chlorophyll is a photosynthetic pigment that participates in light absorption and plays an important role in plant photosynthesis. Since drought stress can accelerate chlorophyll decomposition, chlorophyll content is one of the most commonly used indicators of the severity of drought stress [46,50]. We found that the chlorophyll content of all cultivars significantly decreased under the BF condition. However, cultivars W1 and G1 showed a relatively smaller decline, indicating a stronger ability to maintain their photosynthetic machinery under drought conditions. Bano et al. [51] concluded that a plant maintaining chlorophyll content under drought stress indicates the ability to sustain energy production. However, some studies have also shown that maintaining a lower chlorophyll content under severe drought stress may help plants reduce photo-oxidative damage [52]. This may be related to differences in the plant’s ability to protect chlorophyll from degradation or maintain its synthesis under stress. Plants accumulate reactive oxygen species (ROS) under drought stress, which can impair chloroplast and mitochondrial functions and subject the plant cells to oxidative damage, including lipid peroxidation, protein oxidation, and DNA damage. H_2_O_2_ is one of the ROS closely associated with oxidative stress. It is derived from the disproportionation of superoxide anion, and the product of H_2_O_2_ has a strong oxidation ability [53,54]. Throughout evolution, plants have developed an enzymatic antioxidant system to remove excess ROS, primarily involving ascorbate peroxidase (APX), superoxide dismutase (SOD), peroxidase (POD), and catalase (CAT) [55,56]. Under the SF conditions, cultivars G2, G4, S1, CZ, and others showed a significant increase in antioxidant enzyme activity, indicating a more effective defense against oxidative stress. These cultivars may possess a more efficient antioxidant system, with genes encoding antioxidant enzymes being more sensitive to drought-induced ROS production. Hosseini et al. [57] found that gradual drought reduced the biomass and leaf relative water content of licorice and gradually increased the concentration of osmotic regulatory substances in leaves and the activity of antioxidant enzymes in leaf extracts, which was consistent with the results of this study.

### 3.3. Drought Stress Affects Yield and Quality in C. pilosula Seedlings

Drought stress is a major abiotic stress; the impacts of it on the yield and quality of plants during plant growth and development have been well studied [58,59,60], and they vary based on drought type, intensity, and duration [61,62]. In this study, the water treatment had an impact on the total root length, fresh weight of a single root, and yield of all *C. pilosula* cultivars’ seedlings. In the early stage of drought stress, some plants increase water use efficiency by reducing the lateral growth rate of roots in order to utilize water resources more effectively. Plant roots are usually organs for storing water and nutrients, which directly affect the drought tolerance of plants and the later yield of crops [63]. Under the SF condition, cultivars G1, S1, and CW showed a greater diameter of root compared to the BF condition. This suggests that their root systems have greater water storage and water uptake capacity and will act as a compensatory mechanism when plants face water deficit. Previous studies have shown that efficient root systems have greater root vigor and greater rooting depth, which is a useful trait for improving drought tolerance in crops [64,65]. Meanwhile, cultivars G1, G2, and G4 were able to maintain relatively high yields under the SF condition. This is due to the fact that these cultivars may be more drought-resistant or have undergone long periods of natural selection under drought conditions, resulting in the development of traits adapted to such environments [66].

### 3.4. Comprehensive Evaluation of Different Cultivars of C. pilosula Seedlings Under Drought Stress

The correlation between cultivars and the growth–physiology–yield composite index (GPYCI) reveals that environmental factors have a relatively larger impact on their expression [1]. The drought resistance index is calculated using specific formulas, which take into account various growth and physiological parameters of plants under both the BF and SF conditions [22]. Drought tolerance indices provide a broader and more systematic analysis than analyzing a single growth or physiological indicator, and they contribute to a more in-depth understanding of the mechanisms of drought tolerance in plants as well as the differences in the adaptability of different *C. pilosula* cultivars under drought conditions. Comprehensive factor analysis is a multivariate statistical method. Based on the integrated performance of seedling growth, physiology, and yield of different cultivars under two water conditions, the drought tolerance of different *C. pilosula* cultivars can be assessed more comprehensively. Xiao et al. [67] conducted a comprehensive factor analysis based on six medicinal root trait indicators, early bolting rate, disease resistance, and individual medicinal yield under four fertilization treatments. They ultimately concluded that an organic fertilizer application of 2000 kg/hm^2^ is optimal for the medicinal performance of *Angelica sinensis*. Cui et al. [68] determined through a comprehensive factor analysis of 16 traits of space-grown A. sinensis that the selection efficiency of transplanting space-grown *A. sinensis* 22 h is higher, providing scientific and technical support for the expansion of new cultivars of *A. sinensis*.

## 4. Materials and Methods

### 4.1. The Experimental Area

**Figure 5:**
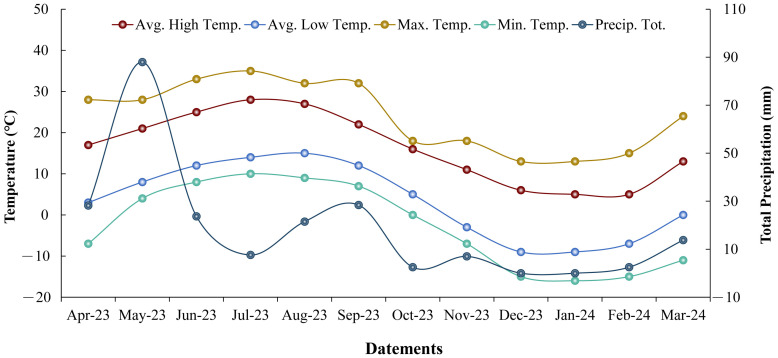
The temperature and rainfall conditions during the experimental period (April 2023–March 2024).

The experiment was conducted from April 2023 to March 2024 in Yuanyi Village, Gongchang Town, Longxi County, Gansu Province (35°01′8″ E, 104°37′11″ N), a traditional area for *C. pilosula* production. Longxi County, located in the Loess Plateau, has a temperate continental climate [69]. The experimental site, at an altitude of 1733.6 m, received an average annual rainfall of 226.3 mm. The temperature and rainfall during the experimental period (April 2023–March 2024) are presented in Figure 5. The previous crop was *Astragalus mongholicus* Bunge, and the field had been fertilized with organic fertilizer for five years, thus creating an organic microenvironment. A composite soil sample was collected using a soil drill with the multi-point composite sampling method from 0 to 20 cm before sowing to evaluate soil nutrients. The loose soil had a soil water content of 203.10 g/kg. Additionally, it showed an electrical conductivity of 192.97 μs/cm and a pH of 7.95 and was enriched with 29.89 g/kg of organic matter. It also contained 29.91 mg/kg of effective phosphorus, 148.23 mg/kg of available potassium, 1.14% of total nitrogen, and had a cation exchange capacity of 12.1 cmol/kg (+).

### 4.2. Experimental Design and Treatments

The field was prepared manually, and the weeds were removed. The experiment adopted a two-factor paired experiment with three replications for each of the nine *C. pilosula* cultivars under normal-watered (black plastic film hole sowing, BF) and water-stressed (Spread in the open field, SF) conditions. The field, covering an area of 224 m^2^, was divided into 54 plots (each with an area of 2.5 m^2^). The seeding density is 3.0 g/m^2^. When broadcasting, the seeds were evenly spread. When sowing in holes on plastic film, the seeding amount per hole is approximately 60 seeds. After sowing, gently cover the seeds with soil, and then cover the entire experimental field with a shade net until the emergence is complete, after which the shade net is removed. The total seeding amount for each plot is 7.5 g, approximately 2500 *C. pilosula* seedlings. Path widths and buffer zones were established to minimize edge effects and facilitate field management and data collection.

The tested cultivars included Weidang No. 1, which is a variety promoted in Dingxi, Gansu Province (the seeds were sourced from the Dry Farming Research and Promotion Center of Dingxi, Gansu Province), and other *C. pilosula* cultivars were selected by the group of Yuan Chen and Feng-xia Guo from Gansu Agricultural University, as detailed in Table 6. The application rate of the organic fertilizer in the experimental field was 270 g/m^2^, which was characterized by an organic matter content of >45.0%, N + P_2_O_2_ + K_2_O ≥ 5.0%, pH within the range of 5.5–8.5, a moisture content of ≤30.0%, and was produced by Gansu Tianyao Biotechnology Co., Ltd., Dingxi City, China. All other field management practices were in line with local large-scale agricultural production.

### 4.3. The Studied Traits

On 10 July 2023, the soil water content of each treatment was determined at intervals of 20 days. Nine cultivars of *Codonopsis pilosula* seedlings were randomly and destructively sampled from each plot to determine growth data. When measuring individual indicators, 20 samples were randomly measured in each replicate block. Leaves were collected on 30 July and 30 August and rapidly frozen with liquid nitrogen for the subsequent evaluation of physiological indexes. After excavation on 14 March 2024, the yield data were determined. According to the local climatic conditions (Figure 5), this study assumed that there was less rainfall (7.6 mm) and a higher temperature in July 2023, creating natural drought stress conditions. The soil water content of each treatment is shown in Figure 1a; there were significant differences in soil water content among the treatments (*p* < 0.05) but no significant differences among the cultivars (*p* > 0.05). The average soil moisture content in the open field plots (SF) for each treatment was 136.5 g/kg, while that in the black plastic film hole sown plots (BF) was approximately 176.1 g/kg. Therefore, the growth data on 10 July and 30 July, the physiological indexes, and the soil moisture content on 30 July were selected for this study to evaluate the drought resistance of *C. pilosula* seedlings of various cultivars.

Soil moisture was determined using the oven-drying method, and the plant height, the number of leaves, the root length, and weight were also measured. The relative growth rate (RGR) was calculated as RGR = (lnHt_2_ − lnHt_1_)/(t_2_ − t_1_), where Ht_1_ and Ht_2_ are the plant heights at times t_1_ and t_2_, respectively [70]. Relative water content (RWC) was estimated according to L. González and M. González-Vilar [71]. The proline levels were assessed with a proline assay kit (A107-1-1, Jiancheng, Nanjing, China). Chlorophyll was determined via ethanol extraction. The antioxidant enzymatic activity was measured at 4 °C [72,73,74].

### 4.4. Drought Tolerance Indices

We identified, in Table 7, the drought tolerance indices used in this study by reviewing other crop-related studies, and we defined stability indices 12–17 based on other formulas.

### 4.5. Statistical Analysis

The results were analyzed via analyses of variance (ANOVAs) and principal component analysis using the SPSS 22.0 software (Chicago, IL, USA). The means were analyzed using the least significant difference (LSD) method at *p* = 0.05 (LSD 0.05). A comprehensive evaluation was conducted according to the methods by Wang et al. [82]. First, based on factor analysis, the principal component values of each indicator whose initial characteristic root was greater than 1 were extracted, and then, the weight (*W_j_*) and positive and negative membership function values were calculated according to the relevant properties. Finally, the comprehensive index (*CI*) was estimated.

*W_j_* = ∑(*C_l,j_* × *VP_l_*)/∑∑(*C_l,j_* × *VP_l_*)

*R*(*X_ij_*) = (*X_ij_* − *X_j_*_min_)/(*X_j_*_max_ − *X_j_*_min_)

*RR*(*X_ij_*) = 1 − (*X_ij_* − *X_j_*_min_)/(*X_j_*_max_ − *X_j_*_min_)

*CI_j_* = ∑[*R*(*X_ij_*) × *W_j_*]

where *C_l,j_* represents the *l*-th principal component of the *j*-th indicator, *VP_l_* is the percentage of the variance of the first principal component, *W_j_* is the weight value of the *j*-th indicator, *i* is different treatment, *j* is the measurement indicator, *R*(*X_ij_*) is the membership function value of indicator *j* under *i* treatment, *RR*(*X_ij_*) represents the value of the anti-membership function of *i* processing the *j*-th indicator, *X_ij_* is the average observation value of indicator *j* of *i* treatment, *X_jmin_* is the minimum value of the *j*-th indicator in all treatments, *X_jmax_* is the maximum value of the *j*-th indicator in all treatments, *CI_j_* is the cumulative composite indicator of the *i*-th indicator. *CI_j_* is the cumulative composite indicator of the *i*-th processed *j* indicator.

## 5. Conclusions

In conclusion, this study evaluated the drought tolerance of different *C. pilosula* cultivars at the seedling stage. The soil environment created was conducive for studying drought resistance. Variations in growth-, physiological-, and yield-related indices were observed among cultivars under different water conditions. The water treatment and the cultivar had different effects on these indices. Cultivar G1 showed better performance in its emergence rate, RGR (number of blades), chlorophyll content, and antioxidant enzyme activities. Cultivar CZ showed better performance in proline content, the content of chlorophyll a/b, and the incidence of disease. Cultivar G2 showed better performance in root diameter, total root length, fresh weight per root, and fresh yield. Drought tolerance indices and comprehensive factor analysis showed that different cultivars had different drought tolerance levels, and a comprehensive evaluation considering multiple indicators was necessary. The comprehensive evaluation ranked the cultivars, with G1 being the most drought-tolerant, followed by CZ, CS, etc. These findings provide valuable information for selecting drought-tolerant *C. pilosula* cultivars in drought-prone areas.

## Figures and Tables

**Table 1:** Analysis of variance for the main and interaction effects of growth indicators.

Source	df	MS/Sig.				
		Emergence Rate	RGR (Plant Height)	RGR (Number of Blades)	RGR (Root Length)	RGR (Single Fresh Weight)
W	1	305.422 **	8.138 × 10^−5^ *	0.000 **	0.002 **	0.000 ns
C	8	56.263 ns	3.240 × 10^−5^ ns	6.704 × 10^−5^ **	2.811 × 10^−5^ ns	7.693 × 10^−5^ ns
Block	2	19.454 ns	0.000 **	1.121 × 10^−5^ ns	0.000 **	0.000 ns
W × C	8	11.319 ns	2.572 × 10^−6^ ns	9.737 × 10^−6^ ns	6.083 × 10^−5^ ns	4.734 × 10^−6^ **
Error	34	36.978	1.647 × 10^−5^	6.393 × 10^−6^	2.754 × 10^−5^	6.584 × 10^−5^

‘W’ means water level; ‘C’ means cultivar; ‘Block’ means replications; ‘Error’ means experimental error; ‘*’ indicates a significant difference (*p* < 0.05); ‘**’ indicates a highly significant difference (*p* < 0.01); ‘ns’ indicates that the difference is not significant.

**Table 2:** Analysis of variance for the main and interaction effects of physiological indicators.

Source	df	MS/Sig.									
		RWC (Leaf)	Proline	APX	POD	SOD	CAT	Chi.a	Chi.b	Chi.a/b	Carotenoid
W	1	5564.354 **	122.949 **	12,072.268 **	14,939.910 **	491.478 **	54,924.322 **	0.266 **	0.031 **	0.043 *	0.021 **
C	8	2.285 ns	31.844 **	10,082.467 **	9004.174 **	380.612 **	26,088.905 **	0.262 **	0.065 **	0.011 **	0.007 **
Block	2	0.635 ns	1.929 ns	3.508 ns	5157.167 *	68.423 ns	663.737 ns	0.000 ns	0.000 ns	0.001 ns	1.993 × 10^−5^ ns
W × C	8	2.577 ns	18.031 **	1199.301 **	600.412 ns	38.736 ns	3868.050 ns	0.022 *	0.004 *	0.017 **	0.002 **
Error	34	12.356	4.566	208.483	1105.257	52.773	3076.853	0.008	0.002	0.001	0.001

‘W’ means water level; ‘C’ means cultivar; ‘Block’ means replications; ‘Error’ means experimental error; ‘*’ indicates a significant difference (*p* < 0.05); ‘**’ indicates a highly significant difference (*p* < 0.01); ‘ns’ indicates that the difference is not significant.

**Table 3:** Analysis of variance for the main and interaction effects of yield and yield-related indicators.

Source	df	MS/Sig.					
		Root Diameter	Total Root Length	Fresh Weight per Root	Total Number of Plants	Yield	Incidence of Disease
W	1	0.215 ns	224.714 **	5.799 **	44.262 ns	83,999,917.780 **	66.168 **
C	8	1.320 **	10.012 ns	1.423 **	61,324.448 **	2,585,914.143 ns	32.410 **
Block	2	0.532 *	3.350 ns	0.263 ns	3484.338 ns	2,171,003.736 ns	3.077 ns
W × C	8	0.362 *	6.618 ns	0.570 *	17,607.675 ns	962,622.148 ns	6.691 **
Error	34	0.141	6.811	0.194	13,991.329	2,377,142.101	1.640

‘W’ means water level; ‘C’ means cultivar; ‘Block’ means replications; ‘Error’ means experimental error; ‘*’ indicates a significant difference (*p* < 0.05); ‘**’ indicates a highly significant difference (*p* < 0.01); ‘ns’ indicates that the difference is not significant.

**Table 4:** Drought tolerance index values of *C. pilosula* cultivar seedlings grown under two water conditions.

Drought Indices	*C. pilosula* Cultivars								
	W1	G1	G2	G4	S1	CW	CS	CZ	CL
*ESI*	0.92	0.88	0.97	0.83	0.98	0.92	0.95	0.89	0.88
*RSI*—Plant height	0.73	0.94	0.98	0.81	0.74	0.85	0.79	0.75	0.59
*RSI*—Number of blades	0.91	0.90	0.93	0.87	0.80	0.79	0.76	0.96	0.64
*RSI*—Root length	0.61	0.79	0.63	0.62	0.41	0.55	0.64	0.67	0.65
*RSI*—Single fresh weight	0.84	0.93	0.96	0.85	0.96	0.98	0.95	0.90	0.88
*RWCSI*	0.76	0.79	0.76	0.79	0.79	0.76	0.77	0.76	0.79
*CSI*	0.95	0.96	0.98	0.93	0.96	0.88	0.74	0.88	0.94
*EASI-CAT*	1.00	0.64	0.97	0.85	0.83	0.21	0.80	0.95	0.64
*EASI-POD*	0.68	0.82	0.90	0.39	0.55	0.86	0.86	0.55	0.69
*EASI-APX*	0.50	0.47	−2.09	−0.35	0.69	0.81	0.86	0.75	0.81
*EASI-SOD*	0.97	0.98	0.98	0.98	1.00	0.97	0.97	0.93	1.01
*PSI*	1.05	1.18	1.02	1.02	1.00	1.13	1.18	1.42	1.04
*STI*	0.43	0.68	0.75	0.76	0.75	0.77	0.74	0.63	0.48
*MPI*	4904.36	6089.67	6600.33	6568.97	6590.75	6648.67	6451.06	5895.11	5182.95
*TOL*	1645.61	1508.67	3531.33	2871.72	3324.17	3262.67	2579.22	1597.11	2129.43
*YI*	0.56	0.73	0.66	0.70	0.67	0.68	0.70	0.69	0.56
*GMP*	48,34.85	6042.77	6359.78	6410.12	6377.73	6445.43	6320.84	5840.77	5072.41
*YSI*	0.71	0.78	0.58	0.64	0.60	0.61	0.67	0.76	0.66
*SSI*	0.85	0.65	1.24	1.06	1.19	1.16	0.98	0.70	1.00
% reduction	28.73	22.04	42.21	35.87	40.28	39.40	33.32	23.86	34.08
*YSSI*	0.64	0.66	1.00	0.91	0.97	0.97	0.86	0.67	0.74
*YPSI*	1629.17	2290.51	1534.25	1848.48	1633.07	1692.80	1935.80	2148.96	1526.49

**Table 5:** Membership value and comprehensive evaluation index of all drought tolerance indices of *C. pilosula* seedlings.

Drought Indices	*C. pilosula* Cultivars								
	W1	G1	G2	G4	S1	CW	CS	CZ	CL
*ESI*	0.600	0.333	0.933	0.000	1.000	0.600	0.800	0.400	0.333
*RSI*—Plant height	0.359	0.897	1.000	0.564	0.385	0.667	0.513	0.410	0.000
*RSI*—Number of blades	0.844	0.813	0.906	0.719	0.500	0.469	0.375	1.000	0.000
*RSI*—Root length	0.526	1.000	0.579	0.553	0.000	0.368	0.605	0.684	0.632
*RSI*—Single fresh weight	0.000	0.643	0.857	0.071	0.857	1.000	0.786	0.429	0.286
*RWCSI*	0.000	1.000	0.000	1.000	1.000	0.000	0.333	0.000	1.000
*CSI*	0.875	0.917	1.000	0.792	0.917	0.583	0.000	0.583	0.833
*EASI-CAT*	1.000	0.544	0.962	0.810	0.785	0.000	0.747	0.937	0.544
*EASI-POD*	0.569	0.843	1.000	0.000	0.314	0.922	0.922	0.314	0.588
*EASI-APX*	0.878	0.868	0.000	0.590	0.942	0.983	1.000	0.963	0.983
*EASI-SOD*	0.500	0.625	0.625	0.625	0.875	0.500	0.500	0.000	1.000
*PSI*	0.119	0.429	0.048	0.048	0.000	0.310	0.429	1.000	0.095
*STI*	0.000	0.735	0.941	0.971	0.941	1.000	0.912	0.588	0.147
*MPI*	0.000	0.680	0.972	0.954	0.967	1.000	0.887	0.568	0.160
*TOL*	0.932	1.000	0.000	0.326	0.102	0.133	0.471	0.956	0.693
*YI*	0.000	1.000	0.588	0.824	0.647	0.706	0.824	0.765	0.000
*GMP*	0.000	0.750	0.947	0.978	0.958	1.000	0.923	0.625	0.147
*YSI*	0.650	1.000	0.000	0.300	0.100	0.150	0.450	0.900	0.400
*SSI*	0.661	1.000	0.000	0.305	0.085	0.136	0.441	0.915	0.407
% reduction	0.668	1.000	0.000	0.314	0.096	0.139	0.441	0.910	0.403
*YSSI*	0.000	0.056	1.000	0.750	0.917	0.917	0.611	0.083	0.278
*YPSI*	0.134	1.000	0.010	0.421	0.139	0.218	0.536	0.815	0.000
Comprehensive evaluation index	0.413	0.783	0.598	0.553	0.597	0.558	0.616	0.603	0.420
Comprehensive sorting	9	1	4	7	5	6	3	2	8

**Table 6:** The details of the experimental cultivars.

Cultivar Number	Cultivar Name	Breeding Methods	Abbreviation
2006-92-02	Weidang No. 1	Pedigree selection	W1
2011-D07-3	Gandang No. 1	Mass selection	G1
2011-D07-1	Gandang No. 2	Mass selection	G2
2011-D07-7	Gandang No. 4	Mass selection	G4
2011-13-3	Space-bred No. 1	Mutation breeding	S1
2011-2-1	Wild patterned *Codonopsis*	Domestication breeding	CW
2011-2-2	Succulent patterned *Codonopsis*	Domestication breeding	CS
2011-1-1	CL	Pedigree Selection	CZ
2011-1-2	CZ	Pedigree Selection	CL

**Table 7:** Drought tolerance indices.

Drought Tolerance Indices		Formula	Reference
1	Stress tolerance index, *STI*	$STI=\frac{{\left(Yi\right)}_{SF}\times {\left(Yi\right)}_{BF}}{\;{\bar{{\left(Yi\right)}_{BF}}}^{2}}$	[1]
2	Mean productivity index, *MPI*	$MPI=\frac{{\left(Yi\right)}_{SF}+{\left(Yi\right)}_{BF}}{2}$	[75]
3	Stress tolerance, *TOL*	$TOL={\left(Yi\right)}_{BF}-{\left(Yi\right)}_{SF}$	[23]
4	Yield index, *YI*	$YI=\frac{{\left(Yi\right)}_{SF}}{{\bar{\left(Yi\right)}}_{BF}}$	[76]
5	Geometric mean productivity, *GMP*	$GMP=\sqrt[{2}]{\;{\left(Yi\right)}_{BF}\times {\left(Yi\right)}_{SF}\;}$	[77]
6	Yield stability index, *YSI*	$YSI=\frac{{\left(Yi\right)}_{SF}}{{\left(Yi\right)}_{BF}}$	[78]
7	Stress intensity, *SI*	$SI=1-\frac{{\bar{\left(Yi\right)}}_{SF}}{{\bar{\left(Yi\right)}}_{BF}}$	[79]
8	Stress susceptibility index, *SSI*	$SSI=\frac{1-\frac{{\left(Yi\right)}_{SF}}{{\left(Yi\right)}_{BF}}}{SI}$	[23]
9	% reduction	$\%\;\mathrm{reduction}=\frac{{\left(Yi\right)}_{BF}-{\left(Yi\right)}_{SF}}{{\left(Yi\right)}_{BF}}\times 100\%$	[80]
10	Yield potential score index, *YPSI*	YPSI=0.5 (MPI+STI)−0.5 (SSI+TOL)	[81]
11	Yield stress score index, *YSSI*	YSSI=0.5 (STI+SSI)	[81]
12	Emergence rate stability index, *ESI*	$ESI=\frac{{\left(Eri\right)}_{SF}}{{\bar{\left(Eri\right)}}_{BF}}$	
13	RWC stability index, *RWCSI*	$RWCSI=\frac{{\left(RWCi\right)}_{SF}}{{\bar{\left(RWCi\right)}}_{BF}}$	
14	Proline content stability index, *PSI*	$PSI=\frac{{\left(Pro.i\right)}_{SF}}{{\bar{\left(Pro.i\right)}}_{BF}}$	
15	Chlorophyll stability index, *CSI*	$PSI=\frac{{\left[\left(Chl.a+b\right)i\right]}_{SF}}{{\bar{\left[\left(Chl.a+b\right)i\right]}}_{BF}}$	
16	RGR stability index, *RSI-X*	$RSI-X=1-\frac{\left|{\left(RGRi\right)}_{SF}-{\left(RGRi\right)}_{BF}\right|}{0.5\;\left[{\left(RGRi\right)}_{BF}+{\left(RGRi\right)}_{BF}\right]}$	
17	Enzyme activity stability index, *EASI-X*	$EASI-X=1-\frac{{\left(Ei-X\right)}_{SF}-{\left(Ei-X\right)}_{BF}|}{{\left(Ei\right)}_{BF}}$	

Where $\bar{{\left(Yi\right)}_{SF}}$ is the mean yield of each cultivar under the water-stressed (*SF*) condition, (*Eri*)*_SF_* is the emergence rate of each cultivar under the SF condition, (*RGRi*)*_SF_* is the relative growth rate of growth parameters of each cultivar under the SF condition, (*RWCi*)*_SF_* is the leaf relative water content of each cultivar under the SF condition, [(*Chl.a* + *b*)*i*]*_SF_* is the chlorophyll content of each cultivar under the SF condition, (*Ei − X*)*_SF_* is the enzyme activity (CAT, APX, POD, and SOD) of each cultivar under the SF condition, (*Pro.i*)*_SF_* is the proline content of each cultivar under the SF condition, (*Yi*)*_SF_* is the yield of each cultivar under the SF condition; $\bar{{\left(Yi\right)}_{BF}}$ is the mean yield of each cultivar under the well-watered (BF) condition, (*Eri*)*_BF_* is the emergence rate of each cultivar under the BF condition, (*RGRi*)*_BF_* is the relative growth rate of growth parameters of each cultivar under the BF condition, (*RWCi*)*_BF_* is the leaf relative water content of each cultivar under the BF condition, [(*Chl.a + b*)*i*]*_BF_* is the chlorophyll content of each cultivar under the BF condition, (*Ei − X*)*_BF_* is the enzyme activity (CAT, APX, POD, and SOD) of each cultivar under the BF condition, (*Pro.i*)*_BF_* is the proline content of each cultivar under the BF condition, and (*Yi*)*_BF_* is the yield of each cultivar under the BF condition.

## Data Availability

Data will be made available on request.

## Appendix A

**Table A1:** The result of principal component analysis of all drought tolerance indices of *C. pilosula* seedlings.

Principal Components	Eigenvalue	Contribution Rate (%)	Cumulative Contribution Rate (%)
1	8.529	38.768	38.768
2	5.175	23.523	62.290
3	2.579	11.722	74.013
4	2.285	10.385	84.398
5	1.642	7.464	91.862

**Table A2:** Capacity and weight of all drought tolerance indices of *C. pilosula* seedlings.

Drought Indices	Load					Weight Value
	1	2	3	4	5	
*ESI*	−0.547	−0.009	−0.168	−0.588	−0.026	0.037
*RSI*-Plant height	−0.334	0.707	−0.426	0.072	0.413	0.057
*RSI*-Number of blades	0.225	0.543	−0.743	0.100	−0.164	0.052
*RSI*-Root length	0.647	0.359	−0.108	0.084	0.526	0.048
*RSI*-Single fresh weight	−0.643	0.466	0.242	−0.370	0.229	0.054
*RWCSI*	0.000	−0.245	0.444	0.773	0.211	0.057
*CSI*	−0.039	−0.365	−0.520	0.443	0.317	0.056
*EASI-CAT*	0.242	−0.099	−0.673	0.138	−0.374	0.048
*EASI-POD*	−0.209	0.225	−0.008	−0.665	0.669	0.061
*EASI-APX*	0.445	−0.096	0.781	−0.191	−0.230	0.051
*EASI-SOD*	−0.406	−0.649	0.244	0.317	0.445	0.061
*PSI*	0.563	0.638	0.145	−0.292	−0.258	0.052
*STI*	−0.699	0.658	0.140	0.228	−0.075	0.045
*MPI*	−0.744	0.615	0.119	0.213	−0.088	0.044
*TOL*	0.991	0.017	0.037	−0.025	0.093	0.023
*YI*	−0.196	0.908	0.18	0.307	−0.022	0.046
*GMP*	−0.684	0.671	0.139	0.230	−0.089	0.046
*YSI*	0.934	0.306	0.066	0.034	0.092	0.031
*SSI*	0.941	0.295	0.051	0.038	0.087	0.030
% reduction	0.940	0.294	0.050	0.044	0.083	0.030
*YSSI*	−0.984	0.077	0.018	0.061	−0.103	0.025
*YPSI*	0.558	0.764	0.196	0.219	0.030	0.045
